# Using a Programme Science approach to substantially reduce the risk of HIV transmission and acquisition in sex transactions among female sex workers in Zimbabwe

**DOI:** 10.1002/jia2.26262

**Published:** 2024-07-10

**Authors:** Frances M. Cowan, Sithembile Musemburi, Primrose Matambanadzo, Phillip Chida, Richard Steen, Rumbidzo Makandwa, Sungai T. Chabata, Albert Takura, Amber Sheets, Raymond Yekeye, Owen Mugurungi, Bernadette Hensen, Joanna Busza, James R. Hargreaves

**Affiliations:** ^1^ Liverpool School of Tropical Medicine Liverpool UK; ^2^ Centre for Sexual Health and HIV AIDS Research Zimbabwe Harare Zimbabwe; ^3^ Population Services International Washington DC USA; ^4^ National AIDS Council Zimbabwe Harare Zimbabwe; ^5^ Ministry of Health and Child Care Harare Zimbabwe; ^6^ Institute of Tropical Medicine Antwerp Belgium; ^7^ London School of Hygiene and Tropical Medicine London UK

**Keywords:** sex workers, HIV prevention, HIV care, Programme Science, programme implementation, Africa

## Abstract

**Introduction:**

We used a Programme Science platform, to generate evidence to support the implementation of programmes for sex workers in Africa. Female sex workers are estimated to make up 1.6% (1.3%–1.8%) of the population of women aged 15−49 years in Zimbabwe. We highlight how programme science can be used to help distinguish between when, where and with whom programmes need to be implemented and discuss two case studies that exemplify implementing better (Case study 1 (1 June 2019−30 June 2021) Optimizing implementation of a risk differentiated microplanning intervention) and implementing differently (Case study 2 (1 October 2016−30 September 2022) Reorientating implementation of DREAMS for young women selling sex).

**Methods:**

Zimbabwe's nationally scaled programme for sex workers was established in 2009 in partnership with sex workers to provide comprehensive services for sex workers and generate evidence for programme design, implementation and scale up. Since inception, comprehensive data have been collected from all sex workers seeking services. As the scope of service provision has expanded so has the scope of data collection and analysis. At enrolment, sex workers are assigned an alphanumeric unique identifier which links consultations within and across programme sites. We conduct descriptive analyses of the Key Population (KP) programme data to guide programme implementation and redesign, embedding programmatic qualitative enquiry as required.

**Results:**

Two case studies describing different approaches to programme optimization are presented. In the first, an optimization exercise was used to strengthen programme implementation ensuring that the KP programme got back on track after SARS‐COV‐2. In the second, an in‐depth review of research and programme data led to a re‐orientation of the DREAMS programme to ensure that young women at the highest risk of HIV acquisition were enrolled and had access to DREAMS social support interventions in turn strengthening their uptake of HIV prevention.

**Conclusions:**

Optimizing and sustaining HIV care and treatment programmes requires effective delivery with sufficient scale and intensity for population impact. Our programme science approach guided the scale up of the KP programme in Zimbabwe, providing evidence to support strategy, implementation and ongoing management, and importantly helping us distinguish between when we needed to just implement, implement better or implement differently.

## 1 INTRODUCTION

Early in the HIV epidemic, programming and research involving female sex workers (FSW) was central to the African response. In the 2000s, the focus shifted to the general population, leaving programmes for sex workers unfunded [1, 2]. A review to identify and characterize FSW programmes across Africa between 2000 and 2011 found only 42 programmes aimed at reaching sex workers and these were largely small, bespoke and integrated into research projects [3]. Additionally, research on sexually transmitted infections and programmes to support their diagnosis and treatment was decoupled from HIV funding [4, 5]. In Asia, however, sex work programming was implemented at scale and associated with demonstrable declines in HIV and sexually transmitted infections (STI) incidence [6, 7, 8, 9, 10, 11].

By the late 2000s, some modelling groups questioned the exclusive focus on general populations in Africa, suggesting that even in generalized epidemics, programming with sex workers for their own benefit would have a broader public health impact, with some models suggesting substantial effects on population‐level incidence [12, 13, 14, 15]. While there was evidence from India of community empowerment approaches having contributed to HIV incidence declines [16], there was little evidence about effective approaches for the very different context of Africa where sex work was less brothel‐based and where there was less history of (and often disincentives to) collectivization.

By the early 2010s, trials demonstrated the effectiveness of new biomedical approaches including treatment for prevention [17] and pre‐exposure prophylaxis [18] but evidence of how to implement them effectively, particularly among hard‐to‐reach populations, was lacking. Additionally, understanding how to ensure sufficient intensity (with saturated programme uptake, and high levels of programme retention and frequency of contacts) as well as at adequate scale (at all identified hotspots of sex work, prioritizing hotspots and individuals at highest risk) to affect population‐level impact was poorly understood. We have used a Programme Science approach in Zimbabwe to address these questions, including identifying need for services, tracking uptake and evaluating implementation strategies for population impact. In order to achieve improved population outcomes, we need to distinguish between when to “just implement,” “implement better” or “implement differently” (see Table 1). We draw on two in‐depth case studies to describe and evaluate our approach.

**Table 1:** The programme science framework in action—an iterative approach to inform planning, implementation and management of the Key Population programme over time

Program Science Framework	*Example*	*Action*
Programme Strategy—demonstrating the ***need for services***	RDS survey data used to construct care cascades in 2011 [19] and 2013 [20] demonstrating high prevalence, poor knowledge of status and in 2013 high levels of viral non‐suppression—but also that young women were more poorly engaged than older women [21]	***Just implement***
Programme Strategy—demonstrating the ***need for services*** and ***whom to target*** for greatest impact	Novel analysis of programme data to illustrate high HIV incidence (which aligned with modelled estimates) and that incidence was higher among younger women than older women [22].	***Just Implement, Implement differently*** *to engage YWSS we developed the Young Sisters model* [23].
Programme Strategy—demonstrating the ***need for services*** and ***where to target*** for greatest impact	Used novel approach to combine data from RDS surveys and programme in 20 sites across Zimbabwe and extrapolate to provide Zimbabwe's first national size estimate for FSW—in so doing identified sites where a high proportion of women were selling sex (>10%) [24].	***Just Implement*** *in more sites*
Programme implementation and management—demonstrating the ***gaps in service*** provision	Novel analysis using RDS data to construct combined condom and PrEP cascade [25] which demonstrated that condom knowledge and supply was good but use was inconsistent and that PrEP supply and adherence was sub‐optimal. This prevention cascade has been widely implemented internationally.	***Implement better*** *—improve demand for condoms and PrEP Strengthen monitoring to optimize use*.
*Case study 1*	*Optimizing implementation of a risk differentiated microplanning intervention to support engagement of sex workers with prevention and care Implementing US Government DREAMS Programme among young women who sell sex*	***Implement better***
*Case study 2*	*Rethinking implementation of the US Government's DREAMS programme for young women who sell sex*	***Implement differently***

Abbreviations: PrEP, pre‐exposure prophylaxis; RDS, respondent‐driven sampling; YWSS, young women who sell sex.

    

## 2 METHODS

### 2.1 Setting and population

#### 2.1.1 Zimbabwe's Key Population Programme

Zimbabwe's nationally scaled programme for sex workers in their diversity is called the Key Populations (KP) Programme (formerly known as “Sisters with a Voice”), run by the Centre for Sexual Health and HIV AIDS Research Zimbabwe (CeSHHAR) on behalf of the Ministry of Health and National AIDS Council over the past 14 years [26]. The KP programme was designed in partnership with sex workers as a Programme Science platform that both provides comprehensive services for sex workers and generates evidence for programme design, implementation and scale up within Zimbabwe and beyond. We conducted a national population size estimate in 2017 that suggested there were 40,491 FSW working in Zimbabwe, 1.23% of women aged 15−49 years, (plausibility bounds 28,177−58,797, 0.86%−1.79%) [27]. By 2023, the KP programme had expanded to over 90 implementation sites in major urban, town and highway hubs, providing extensive community‐based outreach and services.

**Figure 1:**
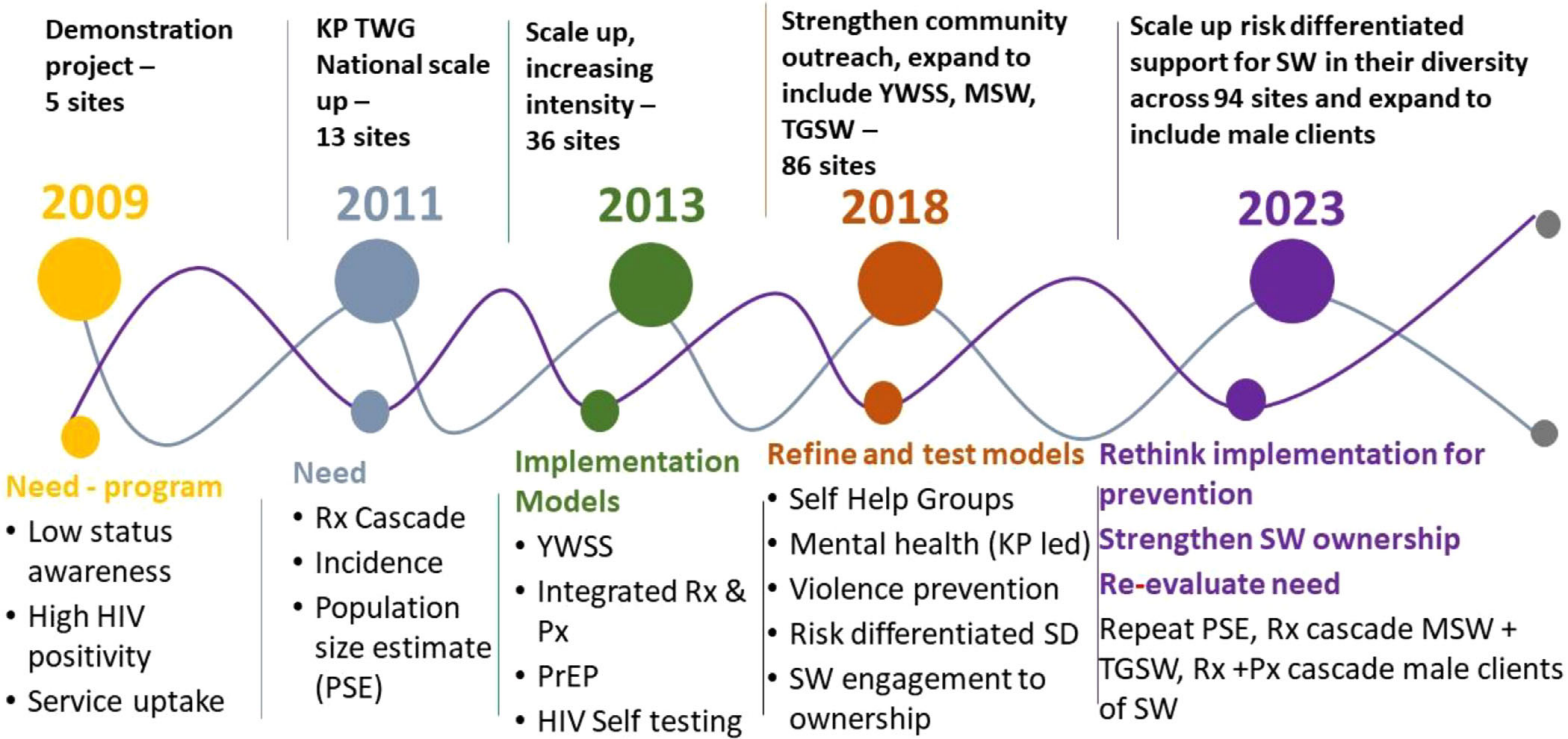
**Zimbabwe Key Population implementation‐research platform**. Abbreviations: KP TWG, Key Populations Technical Working Group; MSW, male sex workers; PrEP, pre‐exposure prophylaxis; Px, prevention; Rx, treatment; TGSW, transgender sex workers; YWSS, young women who sell sex.

As the programme has evolved, it has engaged with different aspects of the Programme Science framework [19], using programme data complemented by targeted quantitative and qualitative research to support strategic planning, and innovative and optimized programme implementation and management (Figure 1). Since 2019, the overall goal has been to substantially reduce the risk of HIV acquisition among HIV‐negative sex workers and to optimize engagement in care among sex workers living with HIV to minimize their morbidity and mortality, thus reducing the risk of onward transmission to sexual partners and children. Here, we present two case studies chosen to illustrate how we worked to identify implementation and how best to strengthen these. In case study 1, programme staff identified that “microplanning” was not being delivered as intended in some sites and provided extra training and supervision to improve delivery. In case study 2, programme staff identified poor implementation mechanisms and designed a different approach in response.

### 2.2 Programme data collection

Since the start of the KP programme, comprehensive data have been collected from all sex workers seeking clinical services and more recently (2016), from sex workers engaged with peer education. Data collection has expanded in line with service provision. At first visit to the KP programme, women are assigned an alphanumeric unique identifier, used to retrieve records at each subsequent visit (CeSHHAR number). The ID links consultations within and across sites, except where women provide incorrect information.

### 2.3 Case study 1—Optimizing implementation of a risk‐differentiated microplanning intervention to support engagement of sex workers with prevention and care—implementing better

Between 2019 and 2021, we conducted an impact evaluation of a programme‐strengthening intervention—AMETHIST (Adapted Microplanning to Eliminate Transmission of HIV in Sex Transactions) that augmented the existing KP programme with peer‐led risk‐differentiated microplanning [20, 21] and self‐help groups [22]. Peer microplanners are FSW considered by their peers to be influential, able to mobilize other FSW and encourage them to take up services. They receive a monthly stipend of US$50 and 5 days training after recruitment. AMETHIST peer‐microplanners took responsibility for a geographic “sex work hotspot,” working to recruit all FSW working in that hotspot (between 50 and 80). They determined each FSW's level of vulnerability every 3 months using a simple risk score and offered follow‐up according to this level, with FSW at high risk being seen weekly, those at moderate risk twice a month and monthly for low risk. They collected data on their caseload, including risk assessments and meetings with each FSW. Alongside clinic attendance, these data were used to measure priority programme indicators (PPIs) and to guide weekly discussions with supervising outreach workers, who reviewed caseloads and discussed challenges with peer‐microplanners and helped them plan the following week's activities.

#### 2.3.1 Setting and population

The AMETHIST trial took place in 22 KP programme sites, randomized 1:1 to the usual KP programme or KP programme augmented by the AMETHIST intervention (11 sites). The intervention targeted FSW who lived or worked in the area surrounding the KP programme site. The primary trial findings are reported elsewhere [23].

#### 2.3.2 Data collection, monitoring and analysis

For this case study, data were compiled from 1 June 2019 to 30 June 2021. Peer‐microplanners mapped the environs surrounding a KP programme clinic every 6 months to identify hotspots of FSW activity. At each hotspot, they estimated FSW numbers on the busiest nights of the month. These data were combined across hotspots to estimate the total number of FSW working in the KP site catchment (*Site level Population Size Estimate* [PSE]).

Each peer‐microplanner took responsibility for FSW working in one hotspot. They tried to recruit all FSW working there to their *hotspot diary*, recording FSW's existing CeSHHAR ID numbers or allocating new ones for FSW not previously seen by the programme. FSW uploaded hotspot diary information into the CeSHHAR microplanning database. These FSW were considered registered with the KP programme; the total number of FSW registered by all peer‐microplanners in a site was defined as **PPI 1**.

Having established rapport with FSW in their caseload, the peer‐microplanner assessed levels of vulnerability using six criteria (age, duration in sex work, drug & alcohol use, condom use, violence and weekly number of clients). This risk score guides the frequency of contacts between peer‐microplanner and FSW (those at high risk [scoring 3−6] seen weekly, moderate risk [scoring 1−3] fortnightly and low risk [scoring 0] monthly). The risk score was developed with FSW and “validated” using survey data [22]. At each contact, the peer‐microplanner provided condoms with support for pre‐exposure prophylaxis (PrEP)/antiretroviral therapy (ART) adherence, and referrals as necessary. Information about each contact was recorded on a “tracking form” (uploaded daily) to guide requirements for next contact and content of supervision meetings between outreach workers and peer‐microplanners.

**PPI 2** is defined as the expected number of contacts (taking account of risk)/PPI 1. Ideally, PPI 2 should be 4 for all FSW thought to be at high risk, 2 for those at moderate and 1 for those at low risk.

Each registered FSW was encouraged by peer‐microplanners to register with the clinic (**PPI 3**—cumulative clinic uptake) and to attend the clinic quarterly for routine check‐ups (**PPI 4**—uptake of quarterly clinic attendance).

PPIs were monitored and discussed in weekly supervision meetings between outreach worker and peer‐microplanner and reviewed monthly at the site level and quarterly across all AMETHIST programme sites.

**Figure 2:**
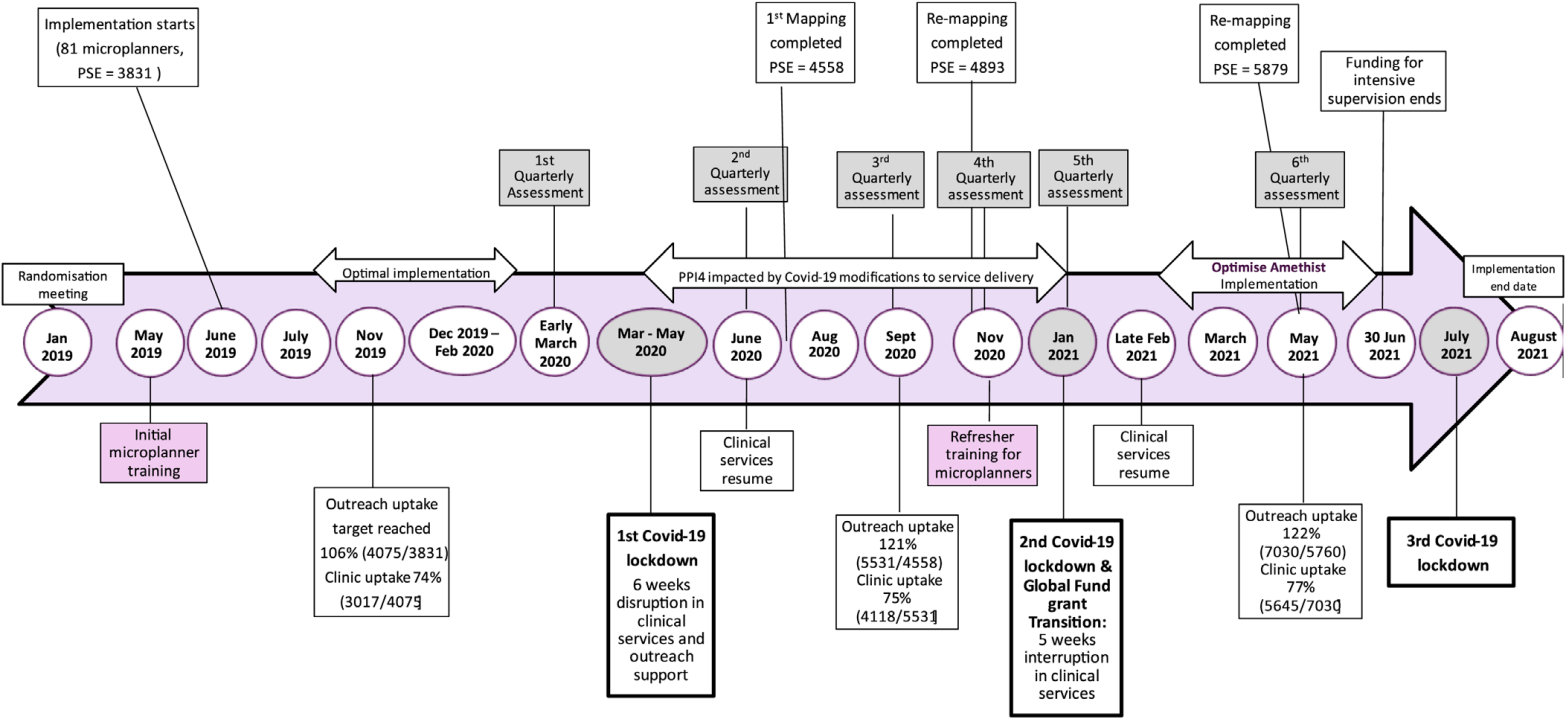
**AMETHIST Implementation Timeline with no mention of PSE**. Abbreviations: PPIs, priority programme indicators; PSE, population size estimate.

Implementation was affected by SARS‐COV2 restrictions (see timeline, Figure 2). When clinical services resumed after the restrictions, we conducted an optimization exercise (the Programme Science focus for this paper) to ensure AMETHIST was being implemented effectively (high‐quality services maximizing opportunity for intervention synergy) with sufficient intensity (uptake saturated, with programme retention and frequency of contacts as intended) and scale (hotspots and individuals at highest risk prioritized).

#### 2.3.3 Optimization

The optimization exercise used microplanning data to identify sites for programme strengthening. It focused on three programme areas (i) getting PPIs back on track after SARS‐COV2; (ii) addressing the HIV testing gap and subsequent linkage to and continuation on PrEP and ART; and (iii) setting sharper epidemiological targets. This exercise was done by site, starting with a review of PPIs, followed by informal discussions among staff and microplanners to identify reasons for gaps in PPIs and strategies to strengthen programme activities, including specifying expected outcomes, identifying who was responsible and by what date they should be achieved.

### 2.4 Case study 2—Reorientating the US Government's DREAMS programme for young women who sell sex in Zimbabwe

In late 2015, the US Government launched the DREAMS (Determined, Resilient, Empowered, AIDS‐free, Mentored and Safe) Partnership in 10 sub‐Saharan African countries, including Zimbabwe [24]. In 2016, the KP programme became one of Zimbabwe's DREAMS Implementing partners providing interventions for adolescent girls and young women who sell sex (AGYWSS) in six districts. Services included HIV testing, referral for oral PrEP or ART as appropriate, contraception, condom provision and “Young Sisters,” group educational sessions designed for AGYWSS [25] with linkage to the broader package of services through referral to other DREAMS implementing partners [24]. We conducted a non‐randomized plausibility evaluation of the impact of DREAMS on HIV incidence among AGYWSS from 2017 to 2018 [28, 29] and found that while access to KP programme clinical services increased, access to socio‐economic interventions promoted by DREAMS remained limited. DREAMS beneficiaries felt this was due to pervasive stigma from non‐KP programme implementing partners (places were often already assigned to lower‐risk girls and young women) as well as entrenched social and material deprivation and structural discrimination hindering their ability to take up the social services offered [30]. In pre‐specified adjusted analysis, HIV incidence was lower in DREAMS sites, although not statistically significant (adjusted RR = 0.68; 95% CI 0.40−1.19; *p* = 0.18) [28].

A broader analysis of DREAMS programme data in Zimbabwe, coupled with the impact evaluation data, led to a rethink of the DREAMS implementation strategy. In 2020, a peer‐administered screening tool was introduced to limit DREAMS enrolment to those at the highest risk of HIV acquisition; all AGYWSS met the screening criteria by definition. Non‐KP implementing partners received training to strengthen KP friendliness. Within the KP programme, an adapted Young Sisters programme was adapted/redesigned to have fewer sessions and completion within 3 months instead of 6. It was renamed “the Girls Club Manual.” Drop‐in centres were established in DREAMS sites (Girls Clubs) and economic strengthening activities were brought in‐house to the KP programme. In 2021, vocational training was also moved within the programme. In 2020, DREAMS implementation expanded from six to nine districts.

#### 2.4.1 Data collection and analysis

For the DREAMS impact evaluation, a cohort of 2400 AGYWSS were recruited across six sites (two implementing DREAMS and four that were not) using respondent‐driven sampling [29]. Cohort recruits were referred to the DREAMS programme in DREAMS sites. The cohorts were followed over 2 years. An in‐depth process evaluation was conducted. All programme indicator data were captured within the DREAMS Zimbabwe database, managed centrally by Population Services International. Data presented here were routine programme data collected between 1 October 2016 and 30 September 2022. All DREAMS enrolees were allocated a unique identifier to allow tracking of enrolees across and between implementing partners and to account for duplicate enrolment. Stakeholders used data from the DREAMS Impact Evaluation to re‐orientate the programme in 2019/20. Anonymized unlinked programme data collected within the DREAMS Zimbabwe database were used to conduct descriptive analysis to illustrate the uptake of various DREAMS activities over time.

### 2.5 Ethics

All analyses presented here use routine programme data. Programme participants do not provide consent for programme participation or programme data collection. Ethics approval for anonymized, unlinked secondary analysis of programme data was obtained from the Medical Research Council of Zimbabwe (MRCZ/E/266).

## 3 RESULTS

### 3.1 Case study 1

The optimization exercise took place in all 11 AMETHIST sites. A mapping exercise was conducted, which found a 17% increase in the number of FSW working across sites (PSE) from 4893 to 5760. Integration of microplanning data and discussion suggested that around 10% of FSW registered with the programme were estimated to be inactive at any time.

**Figure 3:**
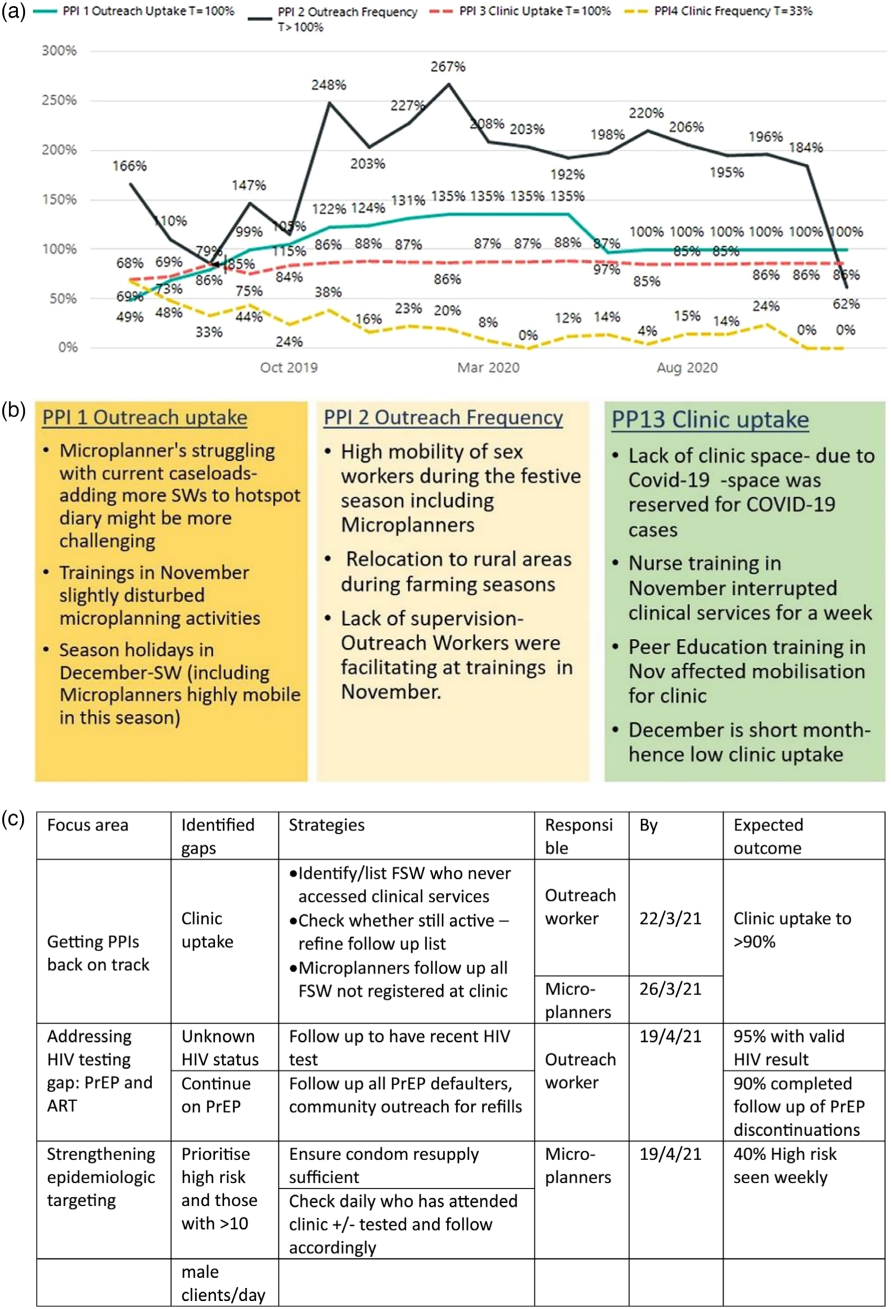
**Design of programme optimization for Site 1**. (a) Review of Priority Programme Indicators (PPIs). (b) Reasons for poor PPI performance at Site 1. (c) Strategies for strengthening performance. Abbreviation: T, target.

Programme data were reviewed in‐depth, sites with a high proportion of young or new entrants to sex work, or where client numbers or violence were high were identified. Where there were gaps in implementation, additional investigation was conducted to understand the underlying reasons. For each site, tailored strategies were developed and time‐bound expected outcomes were selected. As an example, the Site 1 review of PPIs identified several areas of poor performance (see Figure 3a) and the reasons behind these (see Figure 3b); intervention‐strengthening strategies were put in place with regularly monitored outcomes (see Figure 3c). The approach to programme strengthening was tailored to gaps identified at each site.

**Figure 4:**
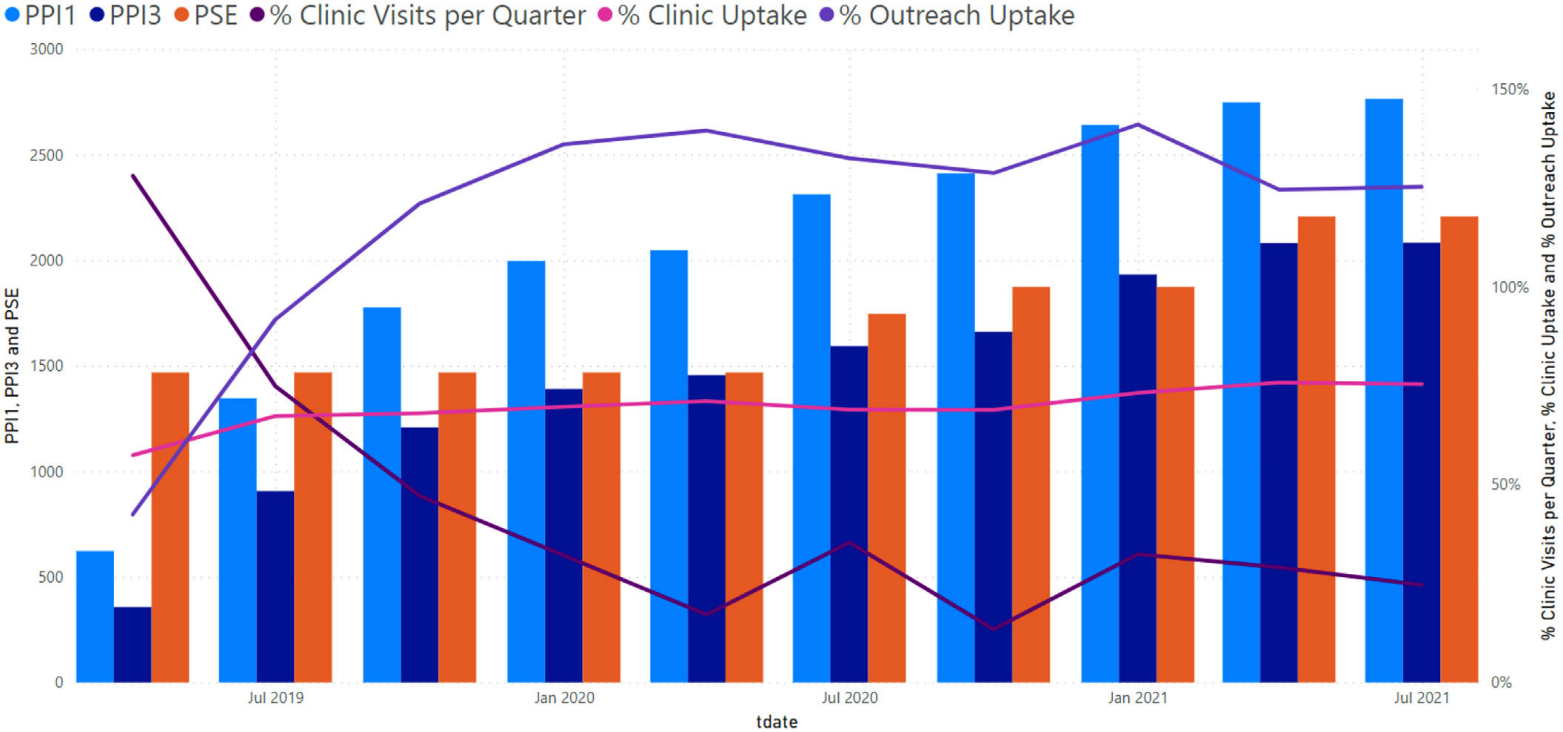
**Impact of optimization on Priority Programme indicators (PPIs)**. Abbreviation: PSE, population size estimate.

The optimization exercise resulted in an increase in both outreach contacts and clinic registrations across the 11 sites as well as monthly clinic visits (Figure 4). Between January 2021 and October 2021, the number of FSW registered with the KP programme increased by 8% to 7142 (although PPI1 apparently decreased, from 135% [*n* = 6606] to 124% [*n* = 7142] overall). While the overall proportion of FSW registered at the clinic remained at 79%, the actual number registered increased from 5219 to 5642 FSW. The proportion of women attending the clinic each month (target 33%) increased from 27% (*n* = 1784) to 34% (*n* = 2428). In addition, some clinical indicators also strengthened across sites (number of HIV tests increased from under 500 Jan−Mar 2021 to around 2000 Aug−Sep 2021; number of FSW newly initiated on PrEP increased from under 100 to over 400 over the same period). Rates of FSW newly identified with HIV and initiated on ART remain high throughout [23]. Of note, the optimization exercise was not conducted in the AMETHIST control sites. While control sites saw a rebound in service uptake after SARS‐COV2 lockdowns, this was lower than in AMETHIST sites [23].

### 3.2 Case study 2

**Figure 5:**
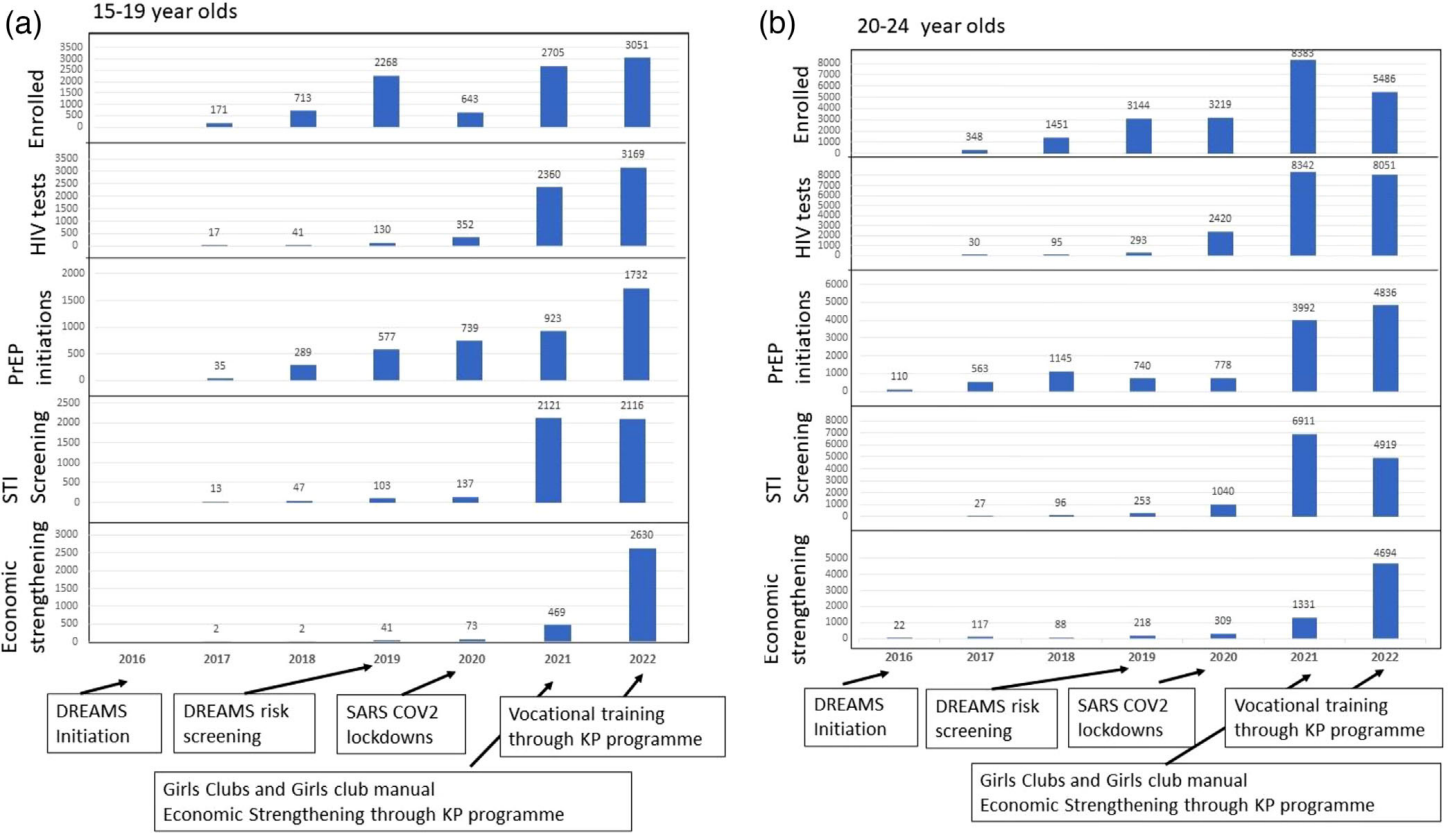
**DREAMS component intervention uptake**. (a) 15‐ to 19‐year old; (b) 20‐ to 24‐year old. Abbreviations: KP, key populations; PrEP, pre‐exposure prophylaxis; STI, sexually transmitted infection.

In the first 3 years of implementation, uptake of clinical services increased but uptake of social interventions was limited (see Figure 5). Specifically, once DREAMS risk screening was introduced, which focused DREAMS enrolments on those at highest risk of HIV, there was an increase in enrolments among AGYWSS. Providing safe spaces (Girls Clubs) and increasing uptake and completion of educational/empowerment programme outlined in the Girls Club Manual coincided with an increase in uptake of clinical services, HIV testing, PrEP initiation and STI testing across all age groups. When the economic strengthening interventions were incorporated within the KP programme, the number of AGYWSS accessing economic strengthening training and joining savings groups increased markedly.

## 4 DISCUSSION

Over the last 14 years, using a Programme Science platform designed in partnership with sex workers, we have embedded research to provide much‐needed evidence to support the implementation of programmes for sex workers in Africa, at sufficient scale and intensity to have a population‐level impact. Specifically, we have conducted novel analyses of programme data [31, 32], triangulated with both survey and qualitative data, initially to shed light on how underserved this population was, then to facilitate strategic development, design and evaluation of programmes and individual programme components. Likely as a result, Zimbabwe has one of the few comprehensive, nationally scaled programmes for sex workers in Africa [26], providing services for around 40,000 FSW annually, while providing a range of insights into programme implementation and its likely impact [32, 33, 34]. Recent data suggest that engagement of FSW in treatment and care is equal to or exceeds that of the general population of adult women in Zimbabwe, with 93% of FSW living with HIV in the AMETHIST intervention arm of the trial being virally suppressed [23], which is counter to the trend seen in other east and southern African countries [34]. Most recently, a combined analysis of programme, survey and modelling data suggests that HIV incidence among FSW may be declining [35]. Here, we have specifically highlighted how Programme Science can be used to help programmes/funders and other stakeholders distinguish between when, where and with whom programmes need to be implemented, as well as whether suboptimal programmes “just” need to be implemented better or whether the approach to programming needs to be re‐thought (and then if appropriate re‐evaluated). We have highlighted two case studies which exemplify implementing better and implementing differently.

In case study 1, we embedded an intervention optimization exercise into a cluster randomized trial of an intervention with the aim of refocusing the intervention after a series of lockdowns associated with SARS‐COV2. This exercise identified site‐specific weaknesses in implementation that was no longer functioning as intended. These weaknesses were then systematically addressed with context‐specific solutions for each setting. For case study 2, an in‐depth review of research and programme data led to a re‐orientation of the DREAMS programme to ensure those at highest risk were enrolled and had access to DREAMS social support interventions in turn strengthening their uptake of HIV prevention.

The strength of our approach is that we have collected comprehensive data from over 140,000 sex workers over time, with programme attendees forming an open cohort, allowing analysis across populations and over time and geographies. We have also conducted a number of qualitative and quantitative research studies often incorporating respondent‐driven sampling surveys through which we have now enrolled over 16,000 women between 2011 and 2023, using similar methods at each survey allowing us to broaden our inferences beyond programme attendees alone, again across populations, time and geographies [26, 36, 37]. Sex workers in Zimbabwe are highly mobile with 60% travelling for work in 2016 (21% travelled frequently domestically and 16% travelled beyond Zimbabwe's borders with the rest travelling less frequently) [38]. As implementation questions arise, we can tailor our approach according to the relevant and available data streams, sometimes opting to use purely routine programme data and other times taking a programme‐driven look at research data either specifically designed for that purpose or using different analytic and triangulation approaches.

This has allowed us to demonstrate that the programme is both needed [39] and has an impact [40, 41]. This was critical at the programme's start when funders were sceptical about the need to work with KP in Africa [1, 42]. Evidence to support programming continues to be required; a recent analysis of the proportion of PEPFAR funding suggested that the proportion of HIV funding allocated for KP programming is lowest in East and Southern Africa [33], despite the recognition that an effective KP response remains critical to maintain the gains made and prevent backsliding [1]. The development of novel analytic approaches has also allowed us to support programme planning and guide implementation. Triangulation of data from programme research and modelling provides novel insights and gives findings added credibility.

There are also limitations to our approach. Although we have measures in place to deduplicate entries to the programme database, this is not complete. Some duplication is both inevitable and justified as people have confidentiality concerns in a country where sex work and other high‐risk behaviours are criminalized and thus provide false identifying information. The extent of duplicate entries is not quantified. For reasons of confidentiality, we have often not been able to link research and programme data directly but have to impute findings from one population to the other. As the programme gets larger and more complex, there is a growing burden of data curation and demand for complex analyses for which funding is often lacking.

As some countries move towards achieving UNAIDS 95‐95‐95, it will become increasingly important that KP programmes are optimized and tailored to local contexts [43]. Ongoing monitoring of sex worker engagement with the treatment cascade will be important both to maintain the gains already made and to identify sub‐populations or geographies where backsliding occurs. Optimizing uptake of prevention has been more challenging [44]. Innovative approaches to improve motivation, opportunity and capability to use prevention effectively are required and will be increasingly important as new prevention technologies emerge.

## 5 CONCLUSIONS

Optimizing and sustaining programmes requires that they are delivered effectively with sufficient scale and intensity for population impact. Our Programme Science approach guided scale up of the KP programme in Zimbabwe, providing evidence to support strategy, implementation and ongoing management, and provided insights into its effectiveness. Importantly, it helped us distinguish between when we needed to just implement, implement better or implement differently.

## COMPETING INTERESTS

None of the authors have any conflict of interest to declare.

## AUTHORS’ CONTRIBUTIONS

FMC wrote the first draft of the manuscript with JH. All authors commenting on drafts. SM, PC, AT and AS were responsible for different aspects of the data management. RS ran the optimization exercise (case study 1) with RM, PM and AT. FC, SM, RM and PM support DREAMS implementation and FMC, SM, STC, AT, AS, BH, JB and JRH were involved in data analysis and optimization of DREAMS. RY and OM oversee the national KP programme on behalf of NAC and MOHCC.

## Data Availability

Selected data are available on request from the authors.
